# A retrospective comparison of CMV DNA detection in reproductive tract secretions *versus* urine: increased yield with combined sample testing

**DOI:** 10.7717/peerj.21197

**Published:** 2026-05-11

**Authors:** Lingwei Hu, Yongying Bai, Peihao Wu, Shuai Li, Kaiqi Wu, Bo Zhu, Yin Binbin

**Affiliations:** 1 Department of Genetics and Metabolism, The Children’s Hospital of Zhejiang University School of Medicine, Hangzhou, China; 2 Department of Clinical Laboratory, The Women’s Hospital of Zhejiang University School of Medicine, Hangzhou, China

**Keywords:** Cytomegalovirus, Reproductive tract secretions, Urine, DNA detection

## Abstract

**Background:**

This study aims to compare cytomegalovirus (CMV) DNA detection rates in reproductive tract secretions (RTS) and urine samples and to further evaluate whether multiple specimen testing improves overall CMV DNA detection rates.

**Methods:**

We conducted a retrospective cohort study involving 3,804 female participants who provided 4,383 samples (10.95% RTS; 89.05% urine) between January 2020 and June 2024. Analyses included: (1) CMV DNA detection rates across sample types and population subgroups, (2) the impact of single versus multiple specimen testing on CMV DNA detection rates

**Result:**

CMV DNA was detected in 3.03% (133/4383) of all samples. Positivity rates differed significantly: 9.79% (47/480) for RTS versus 2.20% (86/3903) for urine (*p* < 0.001). Single-sample testing yielded a positivity rate of 2.46% (82/3,330), while testing multiple specimens increased this to 5.91% (28/474). Concurrent testing of both urine and RTS yielded the highest positivity rate (15.63%, 20/128). Additionally, median CMV viral load was significantly higher in RTS compared to urine (3.34 ± 1.43 *vs*. 0.81 ± 1.47, *p* < 0.001).

**Conclusions:**

The detection rate of CMV DNA in RTS is higher than that in urine. The combined sample testing increases the detection rate of CMV DNA, potentially helping identify women with active viral shedding. These findings inform the development of optimized CMV DNA testing protocols.

## Introduction

Cytomegalovirus (CMV), a member of the Herpesviridae family, is a double-stranded DNA virus. Following primary infection, CMV establishes lifelong latency. When host immune status is altered (*e.g.*, during pregnancy or immunosuppression), latent CMV may reactivate. In China, CMV seroprevalence is notably high, ranging from 90% to 95% (Huang et al., 2024; Wang et al., 2017), exceeding rates reported in many developed countries (Zuhair et al., 2019). Most infected people do not show any symptoms, but newborns can face serious health problems after experiencing a congenital infection (Rawlinson et al., 2017; Ask the Midwife, 2024). Vertical transmission of CMV occurs through three principal routes: transplacental transfer (congenital infection), perinatal exposure during delivery, and postnatal lactation (Hamprecht et al., 2001; Navti, Al-Belushi & Konje, 2021). Therefore, individuals planning to conceive and pregnant women pay particular attention to CMV infection. Given these risks, Chinese clinical guidelines and expert consensus on preconception and antenatal care recommend CMV screening before and during early pregnancy (Ning et al., 2019; Obstetrics Group, Obstetrics and Gynecology Branch of Chinese Medical Association, 2018; Li et al., 2023). Timely identification of high-risk women enables targeted counseling to prevent primary infection during high-risk periods and reduces the occurrence of intrauterine transmission after pregnancy (Navti, Al-Belushi & Konje, 2021; Tanimura & Yamada, 2019).

Currently, clinical detection of CMV relies mainly on detecting specific antibodies in serum. Although this method is helpful for assessing an individual’s past infection history and immune status, it cannot directly reflect viral replication activity or excretion status. CMV testing of non-blood samples can aid in diagnosing and managing infections (Scohy et al., 2024). Detecting the DNA of CMV in non-blood samples (such as urine, saliva, and reproductive tract secretions (RTS)) can provide a method for determining the virus’s excretion status. In contrast to the high seroprevalence, studies detecting active CMV shedding by DNA testing in China have reported much lower, variable positivity rates, ranging from 0.23% to 6.13% across diverse demographic cohorts (Wang et al., 2017; Huang et al., 2021; Zhang et al., 2007; Group for the Study of Maternal and Infantile Cytomegalovirus Infection in Beijing, 2010). Most of these studies, however, have been limited to single-sample testing. Systematic comparisons across sample types, as well as the potential improvement in detection efficacy through combined testing of multiple samples, remain underexplored.

Therefore, to address these gaps, this study aims to compare CMV DNA detection rates in RTS and urine samples and to further evaluate whether concurrent testing of both sample types enhances overall CMV shedding detection.

## Methods

### Participants and materials

**Figure 1:**
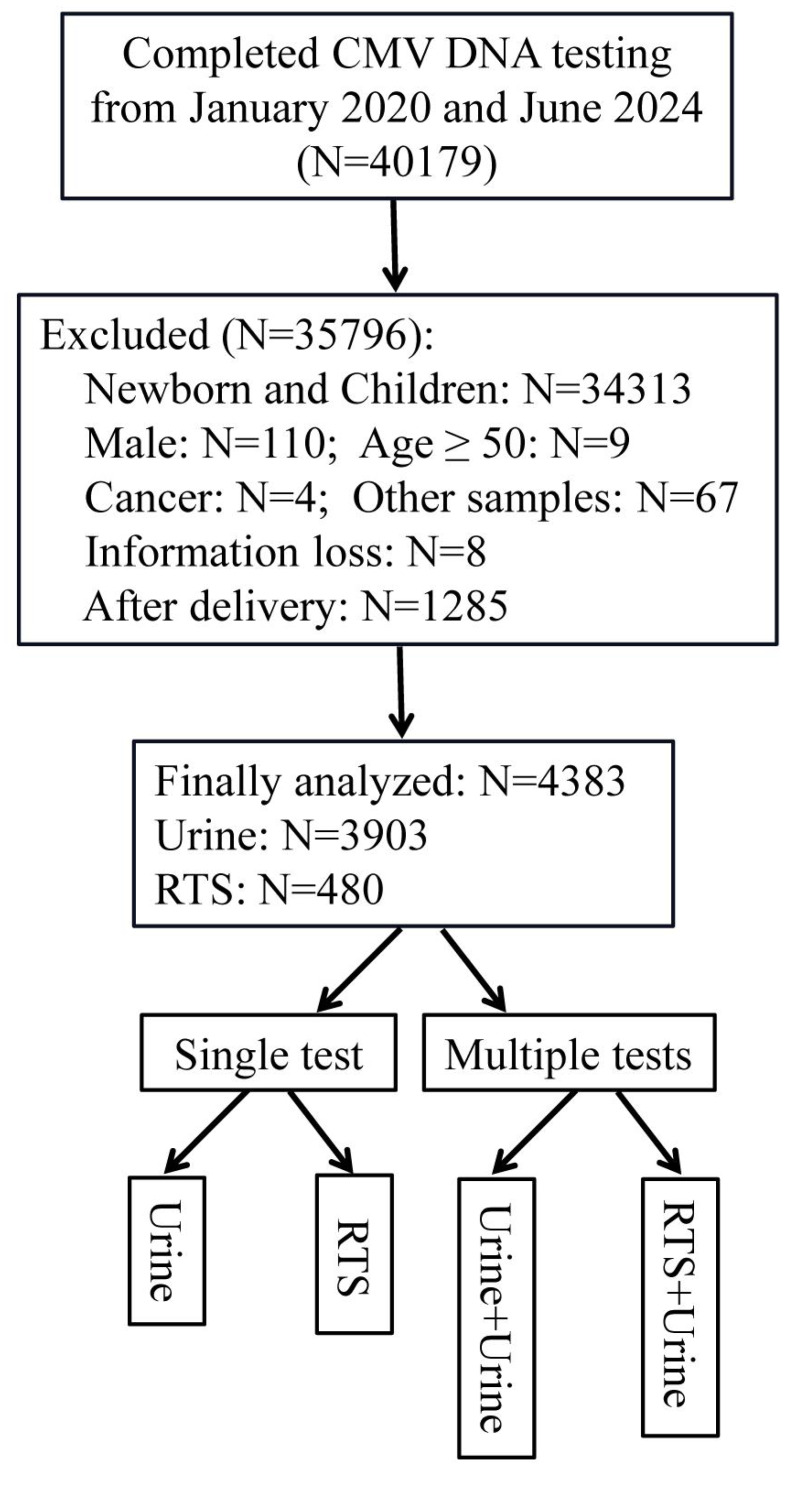
Flow chart of the study design. Other samples: Amniotic fluid 55; Sample type unclear 10; Sputum 2. Abbreviations: CMV, cytomegalovirus; RTS, reproductive tract secretion.

We retrospectively analyzed data from CMV DNA testing performed at the Women’s Hospital of Zhejiang University School of Medicine between January 2020 and June 2024. The study included women who were tested in clinical practice, regardless of their prior CMV serological status. As serological data (IgG/IgM) were not routinely documented in the retrospective dataset, it was not possible to differentiate between primary infection and reactivation. Exclusion criteria: 1. Population: newborns, children, males, and postpartum women; 2. Sample type: amniotic fluid, oropharyngeal samples, or unclear sample type; 3. Women with tumors; 4. Missing information; 5. Women aged ≥ 50 were excluded due to lower fertility and reduced relevance to prenatal screening. Single Testing: Refers to a testing episode where a sample (urine or RTS) from an individual was tested only once during the study period. Multiple Testing: Refers to the scenario where an individual initially testing negative in a first test underwent repeat testing (of the same or different sample type) on two or more subsequent occasions. The overall study design and participant flow are summarized in Fig. 1. Reagents, instruments, and consumables: ABI7500 (Thermo Fisher Scientific, Waltham, MA, USA), CMV Nucleic Acid Detection Kit (PCR-Fluorescent Probe Method) (Daan Gene, Guangzhou, China), 0.9% physiological saline (Kelidai, Shenzen, China), external quality control (BDS, China), sterile swab (CLASSIQSwabs™, Murrieta, CA, USA). This study was approved by the Ethics Committee of the Women’s Hospital, Zhejiang University School of Medicine (IRB-20240353-R) and was conducted in accordance with the ethical principles of the Helsinki Declaration. Informed consent was waived by The Medical Ethics Committee of the Women’s Hospital of Zhejiang University School of Medicine.

### Sample preparation

**Urine:** The urine was centrifuged at 13,523 g for 5 min; the supernatant was discarded, and the remaining sediment was used as a specimen. Add one mL of saline to the residue and mix well, then transfer to an EP tube and centrifuge at 13,523 g for 5 min, discarding the supernatant. To the residue, 50 µL of DNA extraction solution was added, shaken well, incubated at 100 °C for 10 min, then transferred to 4 °C and incubated for 8-12 h. Finally, the mixture was centrifuged at 13,523 g for 5 min to remove debris. The resulting supernatant, containing the extracted DNA, was then used as the template for amplification.

**RTS collection and processing:** RTS samples were gathered from the cervical canal using a sterile swab (CLASSIQSwabs™). The swab head was then placed into a tube containing one mL of sterile saline. The tube was vortexed vigorously to release the material. The liquid was transferred to an EP tube and centrifuged at 13,523 g for 5 min. The supernatant was discarded, and the cellular pellet was processed using the same DNA extraction method as described for the urine sediment (starting with the addition of 50 μL DNA extraction solution).

**Quality control (QC):** For each run, an external quantitative positive control (inactivated CMV culture medium at a known concentration, BDS, China) was processed alongside clinical samples. A volume of 50 μL of the QC was mixed with 50 μL of the DNA extraction solution (lysis buffer provided with the kit) and treated identically to the samples (incubation, centrifugation). The supernatant from this centrifugation step was used for amplification.

### Sample detection and analysis

A highly conserved non-coding region within the CMV IE1 gene was selected as the target for amplification. This region encodes an immediate-early transcriptional regulator, and its high conservation in the reference strain AD169 ensures broad detection specificity. Specific primers and TaqMan fluorescent probes were designed to generate an 86-bp amplicon. Amplification was performed using a commercial kit according to the manufacturer’s protocol. Briefly, the thermal cycling conditions consisted of an initial denaturation at 93 °C for 2 min, followed by 10 cycles of 93 °C for 45 s and 55 °C for 60 s, and then 30 cycles of 93 ° C for 30 s and 55 °C for 45 s. For quantification, a five-point standard curve was generated using serial tenfold dilutions (from 2.00*10^8^ to 1.00*10^4^ copies/mL) of the provided CMV AD169 quantitative standard in saline. A standard curve with a correlation coefficient (R^2^) ≥ 0.97 was required for assay validity. Samples with concentrations calculated from amplification curves below the lower limit of quantification are considered positive, but their quantified values (derived by extrapolation) are indicative rather than precise. Each run included controls: one external positive control, one external negative control, and two no-template controls (blanks) to monitor for contamination and ensure procedural accuracy.

### Statistical analysis

All data were analyzed by SPSS (version 23.0; IBM Corp., Armonk, NY, USA), and *p* < 0.05 indicated statistical significance. To compare differences in CMV DNA copy numbers across samples from the same woman, we used either the matched-samples *t*-test or the Wilcoxon signed-rank test, depending on normality. Data for categorical variables were expressed as numbers and percentages (%), and differences between categorical variables were analyzed using the Pearson *χ*^2^ test.

## Results

### CMV DNA positivity rate of different sample types

**Figure 2:**
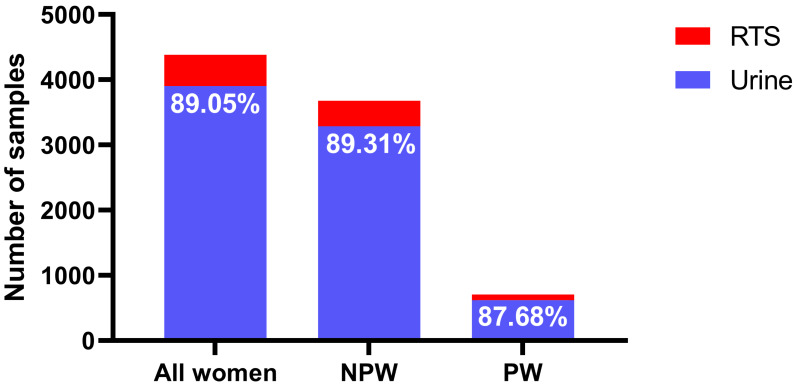
Percentage of urine and RTS in different populations. Abbreviations: NPW, non-pregnant women; PW, pregnant women; RTS, reproductive tract secretion.

**Figure 3:**
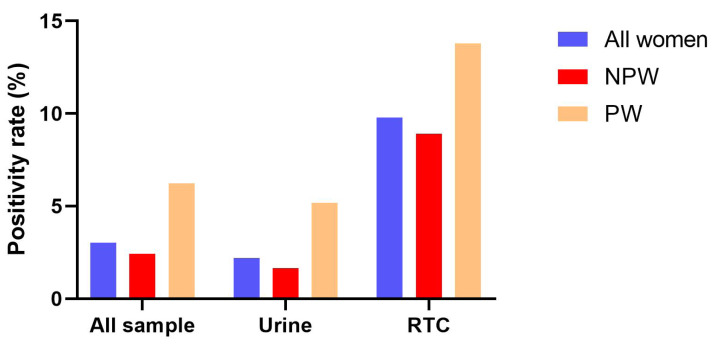
CMV DNA positivity among pregnant and non-pregnant women in different samples. Abbreviations: NPW, non-pregnant women; PW, pregnant women; RTS, reproductive tract secretion.

A total of 4,383 samples were analyzed, among which 706 (16.11%) were from pregnant women, and the remaining 3,677 (83.89%) were from non-pregnant individuals. Urine samples constituted the majority in both groups (87.68% in pregnant and 89.31% in non-pregnant women), with reproductive tract secretions (RTS) comprising the remainder (12.32% and 10.69%, respectively) (Fig. 2). The overall CMV DNA positivity rate was 3.03% (133/4,383). Detection rates differed significantly between sample types: 2.20% in urine *versus* 9.79% in RTS (Fig. 3). Pregnant women exhibited a substantially higher overall positivity rate compared to non-pregnant women (6.23% *vs.* 2.42%, *p* < 0.001). This disparity was consistent across sample types, with positivity rates of 5.17% (urine) and 13.79% (RTS) in pregnant women, compared to 1.64% (urine) and 8.91% (RTS) in non-pregnant women (Fig. 3).

### Impact of testing frequency on CMV DNA detection

**Figure 4:**
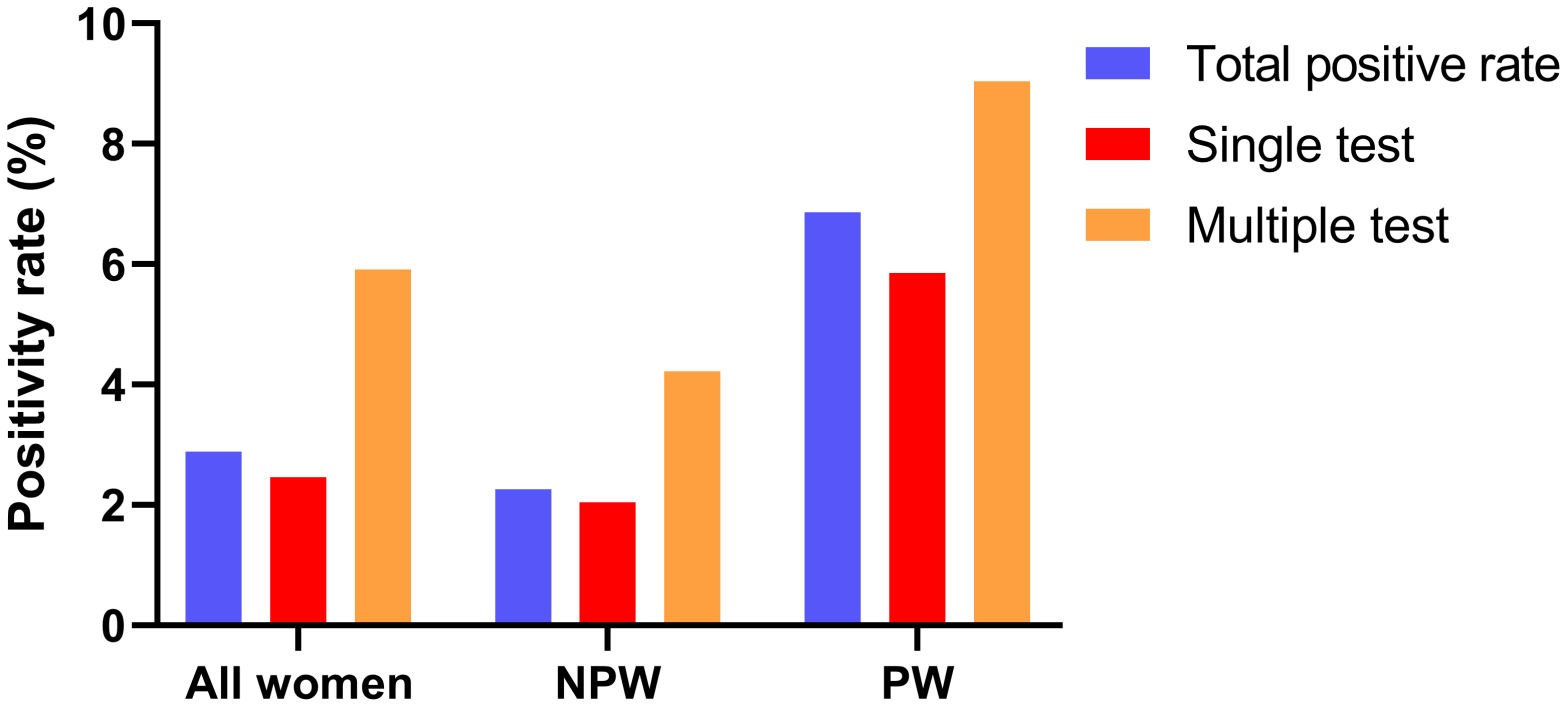
CMV DNA positivity rates in pregnant and non-pregnant women in single and multiple testing. Abbreviations: NPW, non-pregnant women; PW, pregnant women.

The cohort comprised 3,804 women with an overall CMV DNA positivity rate of 2.89% (Fig. 4). Positivity remained significantly higher in pregnant women (6.86%) than in non-pregnant women (2.26%, *p* < 0.001). Most participants (87.54%, 3,330/3,804) underwent single testing (90.12% urine, 9.88% RTS), with an overall detection rate of 2.46% (82/3,330). The rate was 2.05% in non-pregnant and 5.85% in pregnant women within this group. The remaining 12.46% (474/3,804) underwent multiple tests (73.00% serial urine tests; 27.00% combined RTS-urine tests). Multiple testing significantly increased the overall detection rate to 5.91% (*vs.* 2.46% for single testing, *p* < 0.001), with rates of 4.22% in non-pregnant women and 9.04% in pregnant women.

### Comparative efficacy of different testing protocols

**Figure 5:**
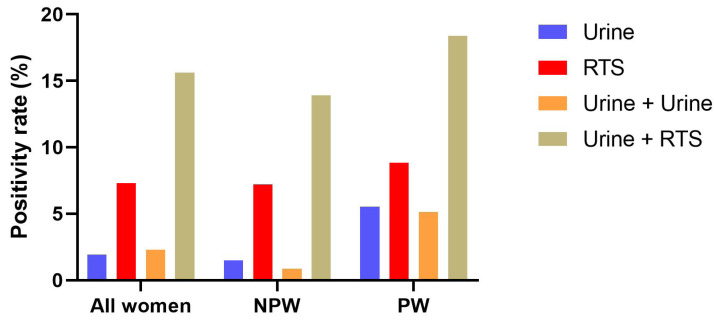
CMV DNA positivity in pregnant and non-pregnant women with different testing protocols. Abbreviations: NPW, non-pregnant women; PW, pregnant women; RTS, reproductive tract secretion.

**Figure 6:**
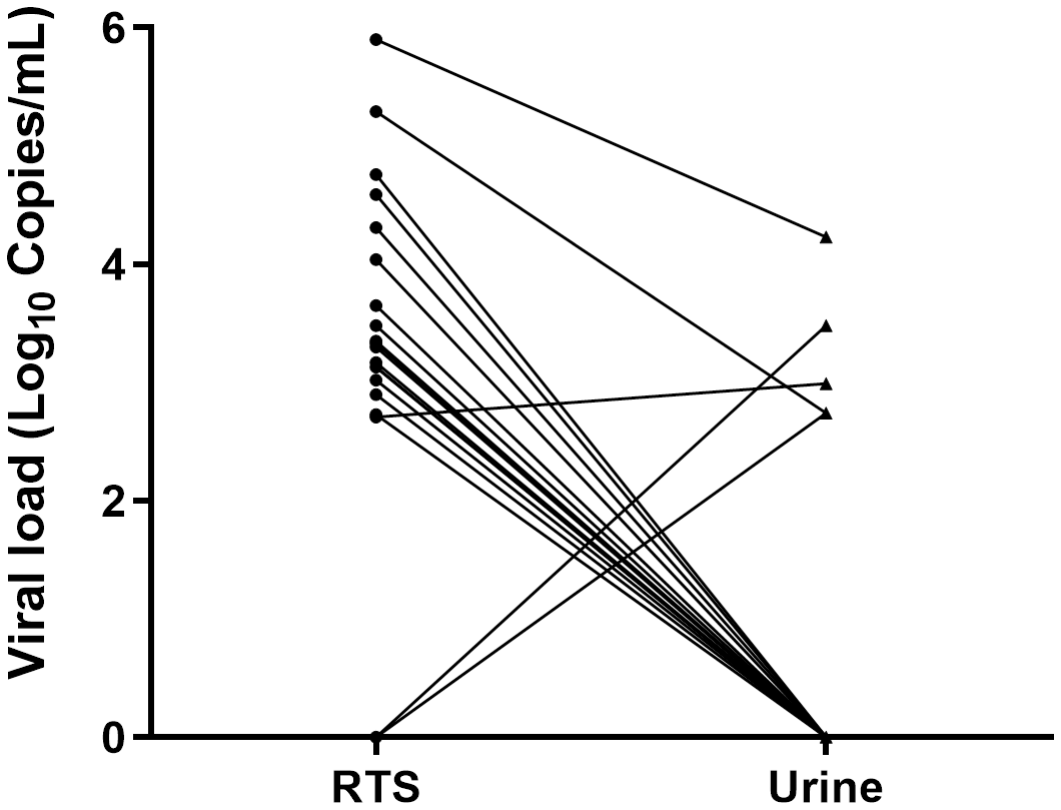
Comparison of CMV viral loads between paired urine and RTS samples (*n* = 20 pairs). Each point represents a sample from an individual. Note that only three individuals had detectable CMV DNA in both sample types. For positive samples with viral loads below 1.00 * 10^4^ copies/mL, the viral load values are estimates calculated by extrapolation of the standard curve. Abbreviations: RTS: reproductive tract secretion.

The CMV DNA positivity rate for single urine testing was 1.93% overall, with rates of 1.49% in non-pregnant and 5.54% in pregnant women (Fig. 5). Single RTS testing yielded a significantly higher overall detection rate of 7.29% (*p* < 0.001 *vs.* single urine), with rates of 7.12% (non-pregnant) and 8.82% (pregnant). Under the multiple-testing protocol, the urine CMV DNA positivity rate was 2.31%, with disparities between subgroups (0.87% non-pregnant *vs.* 5.13% pregnant), though without significant differences compared to single urine testing (*p* = 0.449 and *p* = 0.870, respectively). The highest detection rate was achieved through combined urine-RTS testing, yielding an overall positivity of 15.63% (13.92% in non-pregnant *vs.* 18.37% in pregnant individuals). In a subset of 20 women who provided concurrent urine and RTS samples, the positivity rate was significantly higher in RTS (90.00%, 18/20) than in urine (25.00%, 5/20). Three female samples tested positive in both the RTS and urine assays. The CMV viral load was significantly higher in RTS samples than in urine samples (mean log10 copies/mL ± SD: 3.34 ± 1.43 *vs.* 0.81 ± 1.47, *p* < 0.001) (Fig. 6).

## Discussion

Our study aimed to assess the effects of sample type and testing strategy on CMV DNA detection. We have the following findings: (1) CMV DNA detection rate was significantly higher in RTS compared to urine; (2) Multiple tests and combined tests with multiple samples significantly increased the CMV DNA detection rate; and (3) Pregnant women exhibited a significantly higher rate of CMV DNA detection than non-pregnant women.

Congenital CMV infection may result in permanent neurological damage to the fetus, especially in early pregnancy (Marsico & Kimberlin, 2017; Dollard, Grosse & Ross, 2007). To prevent vertical transmission, early diagnosis and intervention for primary CMV infection during the perinatal period and early pregnancy are essential (Zammarchi et al., 2023; Amir, Chodick & Pardo, 2023; D’Antonio et al., 2023; Chatzakis et al., 2024). The standard method for CMV screening is antibody detection, with seroconversion serving as the diagnostic criterion for primary infection. Relying solely on the CMV IgM test for diagnosis is insufficient due to its limitations, including a narrow detection window, a long incubation period, and the potential for false-positive results (Iijima, 2022; Yin et al., 2023; Leruez-Ville et al., 2020). Primary and non-primary CMV infections can result in the presence of the virus in body fluids, a phenomenon known as CMV shedding (Saldan et al., 2017; Cannon, Hyde & Schmid, 2011). Urine and RTS are frequently used samples for detecting CMV shedding at the molecular level. The detection of CMV antibodies has certain limitations. However, the direct detection of CMV DNA in body fluids provides an additional method for confirming viral shedding. Detecting viral DNA in bodily fluids can also provide direct evidence of active replication in mucosal sites. A key clinical question concerns the relationship between local shedding and systemic viremia, as well as the risk of placental transmission. Our optimized detection strategy aims to identify individuals requiring close monitoring within broader high-risk populations.

This study detected CMV DNA in 3.03% of the samples, with a significantly higher positivity rate in RTS than in urine (9.79% *vs.* 2.20%, *p* < 0.001). Nearly 90% of the samples were urine, and only 10% were RTS. This is because collecting RTS may be uncomfortable for women and increase physicians’ workload. Therefore, women often choose to leave random urine because it is easier to collect. Our study found a higher rate of CMV DNA positivity in RTS and a significant increase in viral load. Previous studies have shown that positivity rates are higher in the RTS than in urine (Ju et al., 2020; Shen et al., 1993; Stagno et al., 1975; Knox et al., 1979; Forman et al., 2017; Sapuan et al., 2022). This result may be due to the following: first, the physician controls the process of collecting RTS, and more cells can be obtained than urine; second, cells of the genital tract may be more susceptible to CMV infection and can act as reservoirs for the virus, releasing it intermittently. The difference in CMV DNA detection rates between pregnant and non-pregnant women is consistent with the understanding that pregnancy is an immunotolerant state. Physiological immune suppression in pregnant women may weaken control over latent viruses such as CMV, leading to increased viral shedding. It has also been noted that the presence of CMV DNA in the RTS can be an predictor of congenital CMV infection in newborns (Tanimura et al., 2017). Therefore, physicians should be concerned that RTS may be more appropriate than urine for detecting CMV shedding and prognostication.

A key innovation of our study was the evaluation of testing frequency and multimodality sampling. Epidemiological investigations in China have reported substantial heterogeneity in the prevalence of congenital CMV infection (Huang et al., 2021; Zhang et al., 2007; Group for the Study of Maternal and Infantile Cytomegalovirus Infection in Beijing, 2010; Wang et al., 2017). The primary reason for this discrepancy is that these studies employed only single-sample designs or single-time-point assessments. Although the above studies used different samples, they did not examine the effects of alternative samples on test results or the impact of combining multiple samples *versus* single-sample testing on results. Our study demonstrated that multiple tests significantly increased the detection rate of CMV DNA (2.46% *vs.* 5.91%, *p* < 0.001). The highest CMV DNA positivity rate of 18.37% was observed in samples sent for combined urine and RTS testing, which was significantly higher than the positivity rates for urine and RTS sent for single testing. Among populations with higher CMV seroprevalence, this is more likely to reflect more frequent, continuous sampling that captures intermittent viral reactivation. This phenomenon is exceptionally reasonable in pregnant women, as changes in their immune status may increase the frequency of reactivation. Therefore, multiple tests with different samples may be considered to improve the detection rate. Although positive results for CMV DNA in urine or RTS samples can confirm viral shedding, such results alone are insufficient to initiate clinical intervention. It is necessary to rely on serological diagnosis of infection, gestational weeks, and fetal ultrasound examination results. Therefore, optimizing detection strategies is crucial for identifying persistent viral shedders, thereby enhancing serological and clinical monitoring to enable timely decision-making when intervention criteria are met.

No previous studies have investigated the impact of multiple testing approaches or different sample types on CMV DNA detection. However, several limitations of our study should be acknowledged. First, the distribution of sample types in this study reflects the actual situation of clinical sample submissions. Urine samples accounted for the majority. Future prospective research designs should focus on collecting paired samples to better evaluate their utility for CMV DNA detection. Second, without knowledge of CMV IgG/IgM status, we cannot determine whether detected viral shedding originated from primary infection or reactivation. Therefore, our results primarily emphasize the greater effectiveness of combined testing in identifying active viral shedding, rather than providing direct guidance for treatment decisions. Third, the unavailability of long-term neonatal outcomes limits our ability to directly correlate specific shedding patterns with congenital infection rates. Future studies should combine serological testing with molecular detection across multiple sample types to comprehensively elucidate the relationships among viral shedding patterns, infection types, and clinical outcomes.

## Conclusions section

In summary, our study found that the detection rate of CMV DNA in the RTS is higher than in urine. A testing strategy that incorporates multiple samples or repeat testing significantly enhances the detection rate, providing a key foundation for optimizing practical CMV DNA screening protocols.

## Supplemental Information

10.7717/peerj.21197/supp-1 Supplemental Information 1 Raw data

## Additional Information and Declarations

### Data Availability

The following information was supplied regarding data availability: The raw data are available in the Supplemental Files.
